# Required muscle mass for preventing lifestyle-related diseases in Japanese women

**DOI:** 10.1186/1471-2458-8-291

**Published:** 2008-08-18

**Authors:** Masae Miyatani, Hiroshi Kawano, Kei Masani, Yuko Gando, Kenta Yamamoto, Michiya Tanimoto, Taewoong Oh, Chiyoko Usui, Kiyoshi Sanada, Mitsuru Higuchi, Izumi Tabata, Motohiko Miyachi

**Affiliations:** 1 Institute of Biomaterials and Biomedical Engineering, University of Toronto, Toronto, Canada; 2 Lyndhurst Centre, Toronto Rehabilitation Institute, Toronto, Canada; 3 Graduate Schools of Human Sciences, Waseda University, Tokorozawa, Japan; 4 Division of Health Promotion, National Institute of Health and Nutrition, Tokyo, Japan; 5 Consolidated Research Institute for Advanced Science and Medical Care, Waseda University, Tokyo, Japan; 6 Department of Sports Health, Matsumoto University, Matsumoto, Japan; 7 Faculty of Sport Sciences, Waseda University, Tokorozawa, Japan

## Abstract

**Background:**

Since it is essential to maintain a high level of cardiorespiratory fitness to prevent life-style related disease, the Ministry of Health, Labour and Welfare of Japan in 2006 proposed to determine the maximal oxygen uptake (*V*o_2_max: mL·kg^-1^·min^-1^) reference values to prevent life-style related diseases (LSRD). Since muscle mass is one of the determinant factors of *V*o_2_max, it could be used as the reference parameter for preventing LSRD. The aim of this study was to determine and quantify the muscle mass required to maintain the *V*o_2_max reference values in Japanese women.

**Methods:**

A total of 403 Japanese women aged 20–69 years were randomly allocated to either a validation or a cross-validation group. In the validation group, a multiple regression equation, which used a set of age and the percentage of muscle mass (%MM, percentage of appendicular lean soft tissue mass to body weight), as independent variables, was derived to estimate the *V*o_2_max. After the equation was cross-validated, data from the two groups were pooled together to establish the final equation. The required %MM for each subject was recalculated by substituting the *V*o_2_max reference values and her age in the final equation.

**Results:**

The mean value of required %MM was identified as (28.5 ± 0.35%). Thus, the present study proposed the required muscle mass (28.5% per body weight) in Japanese women to maintain the *V*o_2_max reference values determined by the Japanese Ministry of Health Labour and Welfare.

**Conclusion:**

The estimated required %MM (28.5% per body weight) can be used as one of the reference parameters of fitness level in Japanese women.

## Background

Previous epidemiologic and clinical evidence indicate that a poor cardiorespiratory fitness is a major risk factor for life-style related diseases (LSRD) such as obesity, hypertension, hypercholesterolaemia, arteriosclerosis and diabetes [1-4]. Moreover, low cardiorespiratory fitness has been found to be a predictor of cardiovascular disease (CVD) mortality, and all-cause mortality [5-8]. Thus, it is essential to maintain a high level of cardiorespiratory fitness to prevent LSRD.

Cardiovascular fitness is usually evaluated as the maximal oxygen uptake per body mass (*V*o_2_max, mL·kg^-1^·min^-1^). The Japanese Ministry of Health Labour and Welfare in 2006 proposed *V*o_2_max reference values for each age group to prevent LSRD [9]. These *V*o_2_max reference values were determined by the "Committee for the Determination of the Recommended Exercise Allowance and Exercise Guide" established in August 2005, and were referenced in the "Exercise and Physical Activity Reference Quantity for Health Promotion 2006 (EPAR2006)". Originally, the "Recommended Quantity of Exercise for Health Promotion (1989)" had been formulated to mainly target the prevention of coronary artery disease. With the passage of more than 15 years following the establishment of this standard, the morbidity pattern of people has worsened and LSRD have increased in prevalence. In order to face this situation, the EPAR2006 was made based on the latest scientific evidence, and was designed to maintain and promote the health of people and prevent LSRD by improving their capacity for physical activity and exercise. These *V*o_2_max reference values proposed in the EPAR2006 were determined by experts through the systematic review of literature regarding the relationship between *V*o_2_max and LSRD such as obesity, hypertension, hypercholesterolemia, diabetes, cerebrovascular disease, CVD mortality and all-cause mortality.

It is well known that *V*o_2_max decreases with age [10-20]. It has been suggested that the age-related decline in *V*o_2_max is a consequence of attenuation of central and peripheral functions such as stroke volume, heart rate max (HR_max_), peripheral O_2 _extraction, and lean body mass (LBM) or muscle mass [19,21-25]. Among these determinants, reductions in HR_max _and LBM or muscle mass have been suggested to be primary factors [26,27]. While many studies on cardiovascular fitness have focused on cardiac measurements, it should be emphasized that muscle mass is one of the critical determinants of *V*o_2_max [13,14,19,24,26,28-30] since the amount of tissue available to extract oxygen during maximal exercise, i.e., muscle, can directly contribute to the value of *V*o_2_max. For example, Sanada et al. reported the MRI-measured lower body skeletal muscle mass was closely associated to the absolute *V*o_2_max during running [28,30]. Additionally, the age-related decrement in *V*o_2_max can be related to the age-associated muscle loss [24,19]. Further, it is important to notice that LBM or muscle mass can be maintained to some degree by exercise training, while such training cannot prevent age-related declines in HR_max_, [26,27].

Therefore, we hypothesized that a certain level of muscle mass required to maintain sufficient cardiovascular fitness is present and that it could be a limiting factor of age-related *V*o_2_max attenuation. Based on this hypothesis, it is advantageous to Japanese women's health to propose such muscle mass required to maintain sufficient *V*o_2_max. Thus, the purpose of this study was to determine a required value of muscle mass to maintain the *V*o_2_max reference value determined by the Japanese Ministry of Health Labour and Welfare in 2006 (Ministry of Health, Labour and Welfare of Japan 2006).

## Methods

### Subjects

A group of 403 Japanese women aged 20 to 69 years were randomly allocated to either a validation group (V-group, n = 201) or a cross-validation group (CV-group, n = 202). The subjects were recruited from the community around the National Institute of Health and Nutrition. All subjects were active and free of overt CVD assessed using a medical history questionnaire. All assessments were conducted at the National Institute of Health and Nutrition between February 2004 and October 2006. The study was approved by the Ethics Committee of the National Institute of Health and Nutrition, and written consent was obtained from all participants.

### Percentage of muscle mass

The lean soft tissue mass of legs and arms were measured with a whole-body Dual Energy X-ray Absorptiomettry (DXA) scanner (Hologic QDR-4500, Hologic INC., Waltham, MA, USA). The body regions were delineated according to specific anatomical landmarks using manual DXA analysis software (version11.2.3). The appendicular lean soft tissue mass was calculated as a sum of the lean soft tissue mass of the legs and the arms. The lean soft tissue mass of extremities assessed using DXA was assumed to represent appendicular skeletal muscle mass along with a small and relatively constant amount of skin and underlying connective tissues. The percentage of muscle mass (%MM) was calculated as follows;

%MM (%) = (Appendicular lean soft tissue mass)/Body weight × 100.

### *V*o_2max_

We assessed peak oxygen uptake (*Vo*_2*peak*_: mL·kg^-1^·min^-1^) instead of *Vo*_2*max *_as an index of cardiorespiratory fitness, which is defined as the highest level of oxygen uptake that is determined by the protocol of a graded exercise load. The *Vo*_2*peak *_was measured using the incremental cycle exercise. An initial work intensity of 30 W or 60 W was selected for each patient based on the patient's fitness level. The work intensity was increased thereafter by a step of 15 W/min, until the subject was not able to maintain the required pedaling frequency of 60 rpm. The heart rate and rating of perceived exertion (RPE) were monitored throughout the exercise. The O_2 _consumption and the minute ventilation were monitored during each 1-min exercise stage (two 30 sec samplings for each stage), after RPE reached 18. The expired air was collected using Douglas bags. Expired O_2 _and CO_2 _gas concentrations were measured using a mass spectrometer (ARCO-1000A, ARCO SYSTEM, Chiba, Japan), and gas volume was measured using a dry gas meter (DC-5C Shinagawa Seiki, Tokyo, Japan). If the subject became exhausted and was not able to keep the pedaling frequency at 60 rpm, it was decided that the maximum effort had been achieved and the test was terminated. The highest value of *Vo*_2 _during the exercise test was designated as *Vo*_2*peak*_. Note that the oxygen uptake obtained in this procedure is referred to as *Vo*_2*peak*_, to discriminate this from *Vo*_2*max *_in the strict definition. However, we equate the obtained *Vo*_2*peak *_to *Vo*_2*max *_in the present study since the *Vo*_2*max *_reference value was determined using both *Vo*_2*max *_and *Vo*_2*peak *_as mentioned in the next section.

### *Vo*_2maxk _reference values

The Japanese Ministry of Health Labour and Welfare proposed *V*o_2_max reference values to prevent life-style related illness for women [9]. The *Vo*_2*max *_reference values are provided for each age group. The procedure to determine *Vo*_2*max *_reference values was described in the EPARQ2006 [9]. In brief, these *Vo*_2*max *_reference values were determined by experts through a systematic review of literature. The target age was 6 years and older. The target LSRD were obesity, hypertension, hyperlipemia, diabetes mellitus, cerebrovascular disorders, death due to circulatory diseases, osteoporosis, ADL and total mortality. By means of this systematic review, the threshold values of the *Vo*_2*max *_or *Vo*_2*peak *_at which the morbidity of LSRD statistically increases in each age group were collected from the literature. The average values of these threshold values for each age group were then calculated and designated as the *Vo*_2*max *_reference values for preventing LSRD. The identified *Vo*_2*max *_reference values (mL·kg^-1^·min^-1^) were 33 (20–29 yr), 32 (30–39 yr), 31 (40–49 yr), 29 (50–59 yr), and 28 (60–69 yr).

### Analyses

First, a single regression analysis was used to test the correlation between age and *V*o_2_max, and between %MM and *V*o_2_max in V-group. Then, a multiple regression analysis was performed using *V*o_2_max as a dependent variable, and age and %MM as the independent variables. This analysis was based on the hypothesis that *V*o_2_max can be accounted for by age and %MM. In this hypothesis, we assumed that the age factor included *V*o_2_max determinant factors related to aging except for muscle mass, such as HR_max_, maximal stroke volume, and peripheral O_2 _extraction [21-23,25,27]. The validity of the prediction by the obtained regression equation was tested by applying the obtained regression equation to the CV-group. After the equation was cross-validated, the data from the two groups were pooled together to obtain the final prediction equation and in the subsequent analysis.

The purpose of the final prediction equation was to obtain the required %MM to maintain the reference *V*o_2_max value in each age group. Thus, the required %MM for each subject was recalculated by assigning the *V*o_2_max reference values and age in the final prediction equation. If the difference of the required %MM among the age groups was very small, the mean value of the required %MM was calculated to be used in the following analysis. To test the validity of the required %MM, the correlation between the sufficiency of *V*o_2_max, i.e., individual's *V*o_2_max as the percentage of the *V*o_2_max reference values (% *V*o_2_max reference values), and the sufficiency of the required %MM, i.e., individual's %MM as the percentage of the required %MM (%required-%MM), were tested.

All data are reported as means ± standard deviations (SD). *P *< 0.05 was used as a level of significance for all comparisons.

## Results

### Physiological characteristics

The physiological characteristics for each group are shown in Table 1. There were no significant physiological differences between V-group and CV-group.

**Table 1:** Characteristics of validation and cross-validation group

	V-group	CV-group
*n*	202	201
Age (yr)	41.4 ± 16.7	41.6 ± 16.9
Height (cm)	158.5 ± 6.4	157.9 ± 6.1
Body weight (kg)	54.4 ± 7.4	53.9 ± 7.3
Body mass index (kg/m^2^)	21.6 ± 2.7	21.7 ± 2.9
Appendicular muscle mass (kg)	16.4 ± 2.4	16.1 ± 2.3
% MMI (%)	30.3 ± 3.2	30.0 ± 3.4
Vo_2_max (ml·kg^-1^·min^-1^)	33.5 ± 7.9	32.7 ± 7.7

mean ± SD, V-group, Validation group; CV-group, Cross-validation group; %MM, percentage of muscle mass

### Relationship between age and Vo_2_max in V-group

**Figure 1:**
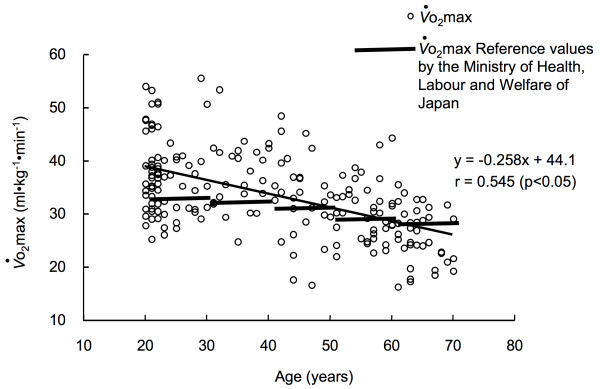
**The relationship between age and *V*o_2_max in the V-group**. The *V*o_2_max reference values by the Japanease Ministry of Health Labour and Welfare were shown for reference.

*V*o_2_max in V-group was from 16.4 to 56.9 ml.kg^-1^min^-1 ^(mean 33.5 ± 7.9) (Table 1). As expected, a strong negative linear correlation was found between *V*o_2_max and age (Figure 1). The decrement was 2.58 ml.kg^-1^min^-1 ^per decade. The *V*o_2_max reference values for each age group in the EPAR2006 were superimposed in Figure 1. With increasing age, the proportion of subjects with *V*o_2_max values below the reference *V*o_2_max values increased.

### Relationship between Vo_2_max and %MM in V-group

**Figure 2:**
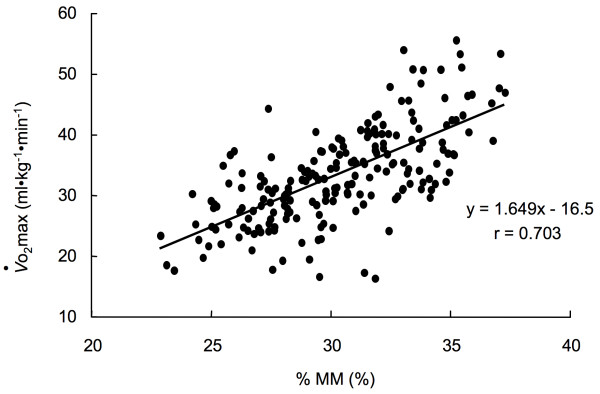
Relationship between percentage of muscle mass (%MM) and *V*o_2_max in the V-group.

%MM in V-group was from 18.7 to 37.3% (mean 30.3 ± 3.2%) (Table 1). There was also a strong correlation between *V*o_2_max and %MM, while the correlation was positive (Figure 2).

### Multiple-regression analysis in V-group

Multiple regression analysis in V-group revealed that age (R^2 ^= 0.286) and %MM (R^2 ^= 0.540) were significant (p < 0.0001) contributors to the prediction of the measured *V*o_2_max. The multiple regression equation obtained in the V-group was the following: *V*o_2_max = -0.135 × Age + 1.315 × %MM -0.799. In this equation, R^2 ^and SEE were 0.522 and 5.4 mL·kg^-1^·min^-1^, respectively.

### Cross-validation of the multiple regression equation

**Figure 3:**
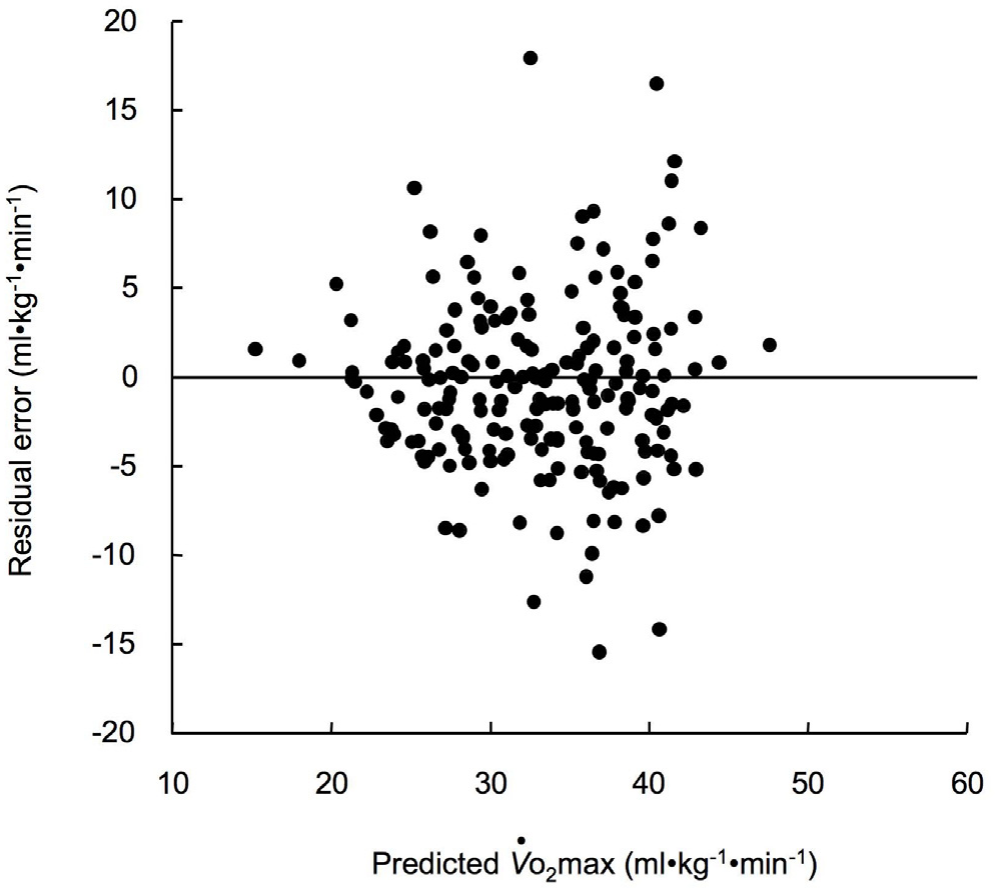
Relationship between estimated *V*o_2_max by the multiple regression equation and the residuals for the CV-group.

The multiple regression equation derived from the V-group was used to predict *V*o_2_max in the CV-group. Figure 3 shows the residual plot. There was not statistically significant correlation between the predicted *V*o_2_max and residual error (p > 0.05). Thus, the residual plot indicates that there was no bias in the prediction of *V*o_2_max of the CV-group using the multiple regression obtained in the V-group.

### Final prediction equation

Data from the two groups were pooled to generate the final equations:

(1) *V*o_2_max = -0.131 × Age + 1.344 × %MM - 2.035.

**Figure 4:**
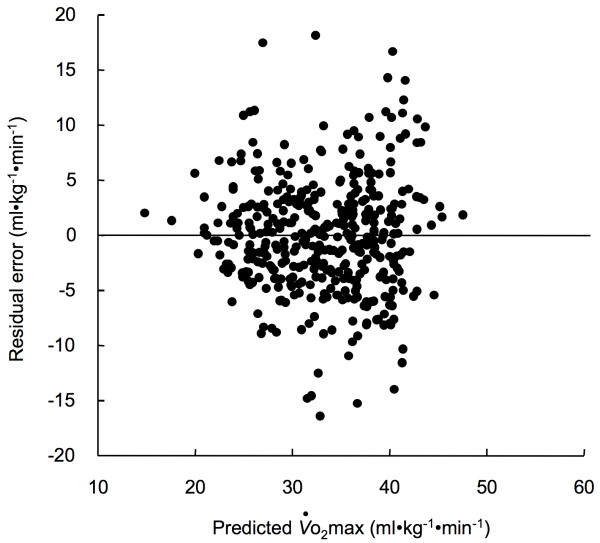
Relationship between estimated *V*o_2_max by the multiple regression equation and the residuals for both the V-group and the CV-group.

In the final equation, analysis revealed that age (R^2 ^= 0.282) and %MM (R^2 ^= 0.570) were significant (p < 0.0001) independent contributors to the prediction of the measured *V*o_2_max. Figure 4 shows the residual plot of the multiple-regression. There was no statistically significant correlation between the predicted *V*o_2_max and residual error (p > 0.05). Thus, the residual plot indicates that there was no bias in the prediction of *V*o_2_max.

### Estimation of the required %MM

The equation (1) was rearranged to predict required %MM as follow;

(2) %MM = (0.131 × Age + 2.035 + *V*o_2_max)/1.344.

**Figure 5:**
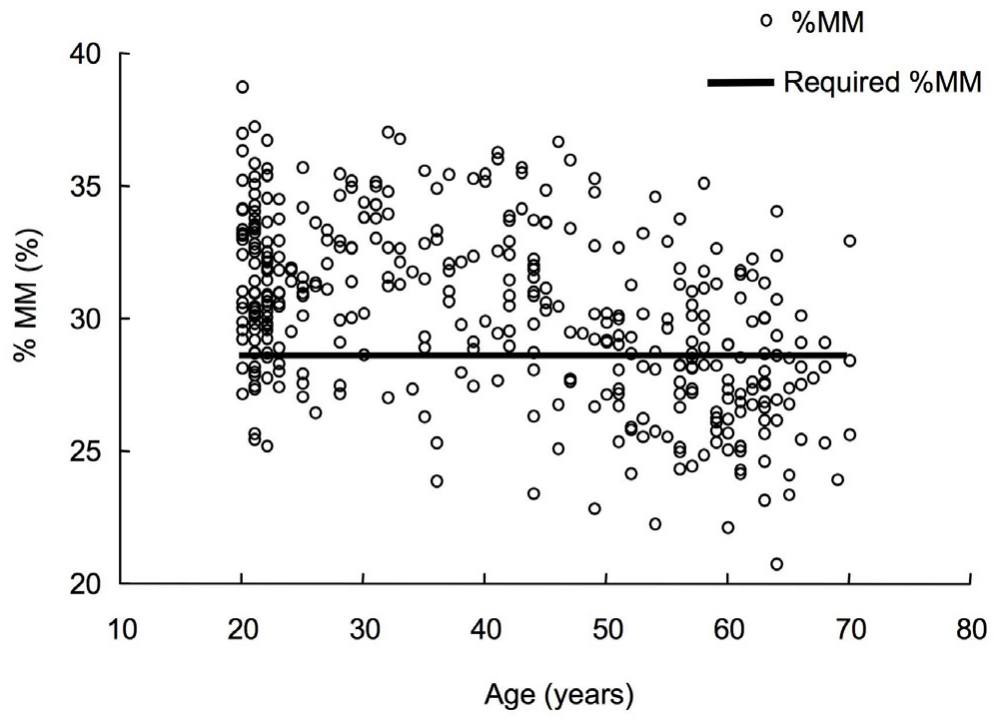
**The relationship between age and the percentage of muscle mass (%MM) in the V-group and the CV-group**. Required %MM is shown for reference.

The required %MM was calculated by assigning the *V*o_2_max reference values, and age in the equation (2). The calculated required %MM was shown in Table 2. The mean value and standard deviation of required %MM was 28.5 ± 0.35%. Figure 5 shows the relationship between the measured %MM and age with the required %MM superimposed on the plot. The older people tended to have a %MM lower than the required. With increasing age, the proportion of subjects with %MM below the required %MM increased.

**Table 2:** Required %MM for *V*o_2_max reference values of each age group

Age group	20 Y	30 Y	40 Y	50 Y	60 Y	Total
*n*	143	48	55	73	84	403
Required MMI (%)	28.3 ± 0.26	28.6 ± 0.29	28.9 ± 0.27	28.4 ± 0.25	28.6 ± 0.30	28.5 ± 0.35

Mean ± SD; %MMI, percentage of muscle mass

### The validity of the required %MM

**Figure 6:**
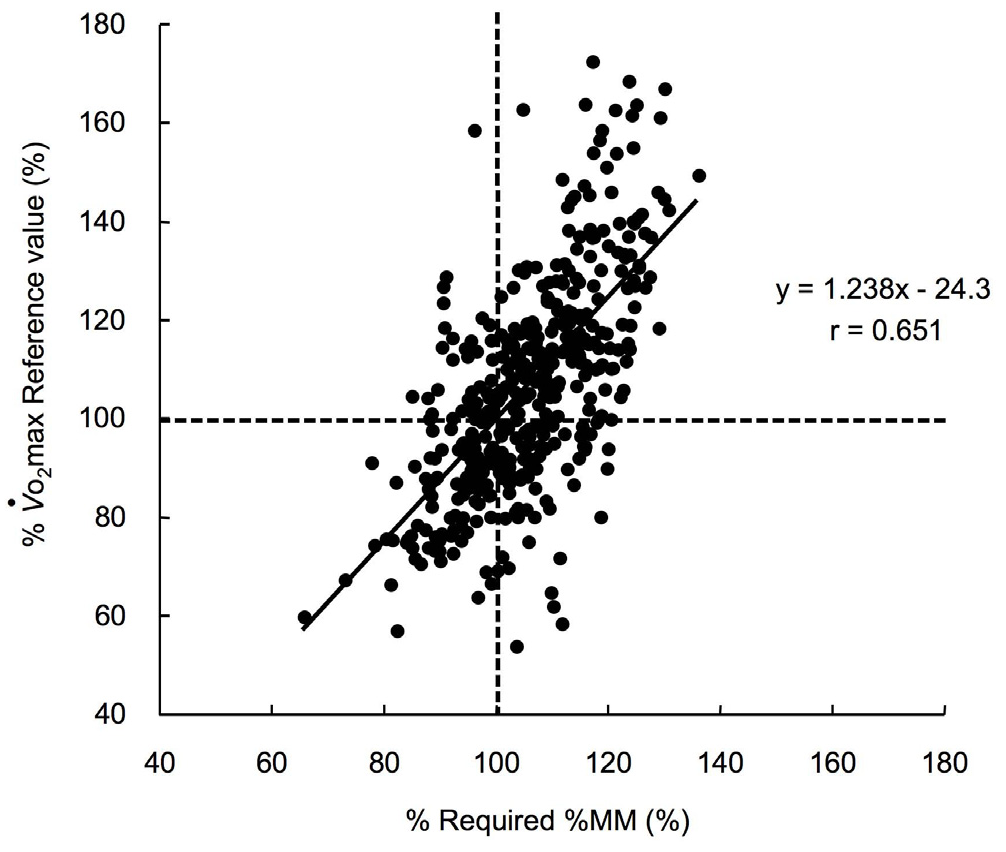
**The relationship between the sufficiency of *V*o_2_max (%*V*o_2_max reference values) and the sufficiency of the required %MM (% required %MM) in both the V-group and the CV-group**. Solid line: regression line, dashed line: lines of 100% of Required %MM and 100% of *V*o_2_max reference values.

Figure 6 shows the relationship between %*V*o_2_max reference values and %required-%MM. The %*V*o_2_max reference values positively correlated with %required-%MM (r = 0.651, p < 0.05).

## Discussion

The primary finding of the present study is that appendicular muscle mass of 28.5% of body weight is needed to maintain the *V*o_2_max reference values determined by the Japanese Ministry of Health Labour and Welfare in Japanese women. By use of the multiple-regression analysis, the regression equation of *V*o_2_max from age and %MM was obtained in the V-group at first. Then the validity of the regression equation was confirmed in the CV-group (Figure 3). The required %MM to maintain the *V*o_2_max reference values was obtained using the final regression equation using the data of V- and CV-groups (equation (2)) and the *V*o_2_max reference values for each age group (Table 2). There was strong correlation between percentages of the required %MM and *V*o_2_max reference values (Figure 6).

### Required muscle mass

We propose the required %MM in Japanese women as a reference value of muscle mass for the usage of maintaining the reference value of *V*o_2_max proposed by the Ministry of Health Labour and Welfare of Japan. Interestingly, the calculated required %MM was not different among age groups (Table 2). Thus, we proposed the averaged required muscle mass (28.5%) as the general value for all age groups. A large portion of the subjects (68%) satisfied the required muscle mass, while with increasing age, the proportion of subjects with %MM below the required %MM increased (Figure 5). This tendency was similar to *V*o_2_max, i.e., with increasing age, the proportion of subjects with *V*o_2_max values below the reference *V*o_2_max values increased (Figure 1). Additionally, there was strong positive relation between percentages of *V*o_2_max reference values and required %MM (Figure 6). The results indicate that subjects with total muscle mass lower than 100% of the required %MM also tended to have lower *V*o_2_max when compared to levels of *V*o_2_max reference values. Thus, our result suggests that one of the reasons for insufficient *V*o_2_max may be insufficient %MM. Women who have %MM less than the required %MM are encouraged to increase their %MM above the required %MM to achieve the *V*o_2_max reference values. The required %MM can be used as an additional parameter for preventing LSRD together with the *V*o_2_max reference values. The required %MM obtained in this study is practical and appropriate for most Japanese women, because it is slightly less than the average %MM of the total number of subjects. Thus, the value is an achievable goal for most of Japanese women. Although strength training is not typically included in exercise programs targeting prevention of the age-related decline in *V*o_2_max or to increase *V*o_2_max, it would be advisable to recommend some form of strength training as well as aerobic training especially for individuals who do not achieve the required %MM.

Several prior studies demonstrated the significance of fat free mass, muscle mass, and/or muscle function to morbidity and mortality, although there are few researches targeting women [31-33]. The Japanese Ministry of Health Labour and Welfare also has admitted the importance of muscle mass and muscle function to prevent LSRD and/or mortality in EPAR2006. However practical target values have not been offered in the statement due to the lack of evidences compared to *V*o_2_max. In this present study we determined the target value of muscle mass through the *V*o_2_max reference values, which already has strong evidences. Although we have not confirmed the direct relation between muscle mass and LSRD morbidity and/or mortality, we believe Japanese women could aim to achieve the required %MM as one of targets for their health. Whether an increase of skeletal muscle mass would result in an improvement of exercise capacity and or reduce morbidity and mortality needs to be confirmed by future studies.

It should note that some individuals may have a large muscle mass, yet be at a high mortality risk. For example, it is well known that central obesity is one of risk factor of LSRD morbidity. Thus, it is important to remember that muscle mass is not the only important parameter but also, other risk factor should be monitored and considered together.

### Prediction of Vo2max from age and muscle mass

The residuals of the multiple regression might be due to the approximation that all age-related determinant factors were included in age in the multiple regression. In the present model, we hypothesized that determinants such as HR_max_, maximal stroke volume, and peripheral O_2 _extraction were age-related, and therefore their effects were included in the factor of age. It was suggested that HR_max _[14,22,26,29,34-39] and peripheral O_2 _extraction [21,34] do decline with age, and are not influenced by exercise training. However, although maximum stroke volume was also suggested to decline with age in sedentary individuals [23], it was suggested that age-related decline of maximum stroke volume was prevented by exercise [21,34]. Thus, the simplification must be the error factor, and it is likely in future to improve the multiple regression equations using these age-related *V*o_2_max determinants, and to improve the estimation of the required MMI.

We studied only a statistical relationship between *V*o_2_max and muscle mass. Therefore, the results do not necessarily suggest a cause-effect relationship. It is possible that muscle mass and *V*o_2_max are physiologically unrelated but indirectly correlated, i.e., people with a high *V*o_2_max may be more physically active and perform activities that increase muscle mass. However, muscle mass is highly likely physiologically important determinant of *V*o_2_max because the amount of tissue available to extract oxygen during maximal exercise directly contribute to the value of *V*o_2_max.

### Study limitations

The current study has limitations that require caution when interpreting and generalizing the findings reported herein. This study included only the cross-sectional design, and it did not investigate the relationship between the required %MM and the morbidity of LSRD or mortality by using a prospective design. Thus, it has not been clarified how the required %MM reflects these risks in this present study. Further investigation is required to validate the required %MM through a prospective study with the morbidity and/or mortality as an endpoint. Additionally, the potential difference between methods using %MM or absolute muscle mass (kg) as the indicator of health should be also investigated. Another limitation of this study is the results of this study are applicable to only Japanese women. The decided %MM in this study may not be able be applicable to men and/or other racial group since they may have different characteristics of the relationship between muscle mass and *V*o_2_max.

## Conclusion

In conclusion, the present study proposed the required muscle mass (28.5% per body weight) in Japanese women to maintain the *V*o_2_max reference values determined by the Japanese Ministry of Health Labour and Welfare. This required muscle mass can be used as one of the reference parameters of fitness level in Japanese women.

## Competing interests

The authors declare that they have no competing interests.

## Authors' contributions

MM performed analysis and data interpretation as well as drafted and revised the manuscript. KM participated in the conception of this study, interpretation of the analysis and critically reviewed this manuscript, and provided comment as Statistical expertise. HK, YG, KY, MT, TO, CU and SK performed data analysis and interpretation, and provided comment and review of the manuscript. MH and IT designed the project, assisted with data interpretation and provided comment and revisions for the manuscript. MM designed the project, participated in the conception of this study, interpretation of the analysis and critically reviewed this manuscript. All authors read and give final approval of the final manuscript for publication.

## Pre-publication history

The pre-publication history for this paper can be accessed here:



## References

[B1] Sawada S, Tanaka H, Funakoshi M, Shindo M, Kono S, Ishiko T (1993). Five year prospective study on blood pressure and maximal oxygen uptake. Clin Exp Pharmacol Physiol.

[B2] Sawada SS, Lee IM, Muto T, Matuszaki K, Blair SN (2003). Cardiorespiratory fitness and the incidence of type 2 diabetes: prospective study of Japanese men. Diabetes Care.

[B3] Lakka TA, Laukkanen JA, Rauramaa R, Salonen R, Lakka HM, Kaplan GA, Salonen JT (2001). Cardiorespiratory fitness and the progression of carotid atherosclerosis in middle-aged men. Ann Intern Med.

[B4] Wei M, Gibbons LW, Mitchell TL, Kampert JB, Lee CD, Blair SN (1999). The association between cardiorespiratory fitness and impaired fasting glucose and type 2 diabetes mellitus in men. Ann Intern Med.

[B5] Blair SN, Kampert JB, Kohl HW, Barlow CE, Macera CA, Paffenbarger RS, Gibbons LW (1996). Influences of cardiorespiratory fitness and other precursors on cardiovascular disease and all-cause mortality in men and women. JAMA.

[B6] Wei M, Kampert JB, Barlow CE, Nichaman MZ, Gibbons LW, Paffenbarger RS, Blair SN (1999). Relationship between low cardiorespiratory fitness and mortality in normal-weight, overweight, and obese men. JAMA.

[B7] Myers J, Prakash M, Froelicher V, Do D, Partington S, Atwood JE (2002). Exercise capacity and mortality among men referred for exercise testing. N Engl J Med.

[B8] Laukkanen JA, Lakka TA, Rauramaa R, Kuhanen R, Venalainen JM, Salonen R, Salonen JT (2001). Cardiovascular fitness as a predictor of mortality in men. Arch Intern Med.

[B9] Ministry of Health LaWoJ (2006). Exercise and physical activity reference quantity for health promotion 2006 (EPARQ2006) – Physical Activity, Exercise, and Physical Fitness.

[B10] Astrand I (1960). Aerobic work capacity in men and women with special reference to age. Acta Physiol Scand Suppl.

[B11] Drinkwater BL, Horvath SM, Wells CL (1975). Aerobic power of females, ages 10 to 68. J Gerontol.

[B12] Hossack KF, Bruce RA (1982). Maximal cardiac function in sedentary normal men and women: comparison of age-related changes. J Appl Physiol.

[B13] Toth MJ, Gardner AW, Ades PA, Poehlman ET (1994). Contribution of body composition and physical activity to age-related decline in peak VO2 in men and women. J Appl Physiol.

[B14] Jackson AS, Wier LT, Ayers GW, Beard EF, Stuteville JE, Blair SN (1996). Changes in aerobic power of women, ages 20–64 yr. Med Sci Sports Exerc.

[B15] Tanaka H, Desouza CA, Jones PP, Stevenson ET, Davy KP, Seals DR (1997). Greater rate of decline in maximal aerobic capacity with age in physically active vs. sedentary healthy women. J Appl Physiol.

[B16] Schiller BC, Casas YG, Desouza CA, Seals DR (2001). Maximal aerobic capacity across age in healthy Hispanic and Caucasian women. J Appl Physiol.

[B17] Wiswell RA, Hawkins SA, Jaque SV, Hyslop D, Constantino N, Tarpenning K, Marcell T, Schroeder ET (2001). Relationship between physiological loss, performance decrement, and age in master athletes. J Gerontol A Biol Sci Med Sci.

[B18] Buskirk ER, Hodgson JL (1987). Age and aerobic power: the rate of change in men and women. Fed Proc.

[B19] Fleg JL, Lakatta EG (1988). Role of muscle loss in the age-associated reduction in VO2 max. J Appl Physiol.

[B20] Stathokostas L, Jacob-Johnson S, Petrella RJ, Paterson DH (2004). Longitudinal changes in aerobic power in older men and women. J Appl Physiol.

[B21] Rivera AM, Pels AE, Sady SP, Sady MA, Cullinane EM, Thompson PD (1989). Physiological factors associated with the lower maximal oxygen consumption of master runners. J Appl Physiol.

[B22] Heath GW, Hagberg JM, Ehsani AA, Holloszy JO (1981). A physiological comparison of young and older endurance athletes. J Appl Physiol.

[B23] Hagberg JM, Allen WK, Seals DR, Hurley BF, Ehsani AA, Holloszy JO (1985). A hemodynamic comparison of young and older endurance athletes during exercise. J Appl Physiol.

[B24] Proctor DN, Joyner MJ (1997). Skeletal muscle mass and the reduction of VO2max in trained older subjects. J Appl Physiol.

[B25] Weiss EP, Spina RJ, Holloszy JO, Ehsani AA (2006). Gender differences in the decline in aerobic capacity and its physiological determinants during the later decades of life. J Appl Physiol.

[B26] Hawkins SA, Marcell TJ, Victoria Jaque S, Wiswell RA (2001). A longitudinal assessment of change in VO2max and maximal heart rate in master athletes. Med Sci Sports Exerc.

[B27] Hawkins S, Wiswell R (2003). Rate and mechanism of maximal oxygen consumption decline with aging: implications for exercise training. Sports Med.

[B28] Sanada K, Kearns CF, Kojima K, Abe T (2005). Peak oxygen uptake during running and arm cranking normalized to total and regional skeletal muscle mass measured by magnetic resonance imaging. Eur J Appl Physiol.

[B29] Jackson AS, Beard EF, Wier LT, Ross RM, Stuteville JE, Blair SN (1995). Changes in aerobic power of men, ages 25–70 yr. Med Sci Sports Exerc.

[B30] Sanada K, Midorikawa T, Yasuda T, Kearns CF, Abe T (2007). Development of nonexercise prediction models of maximal oxygen uptake in healthy Japanese young men. Eur J Appl Physiol.

[B31] Allison DB, Zhu SK, Plankey M, Faith MS, Heo M (2002). Differential associations of body mass index and adiposity with all-cause mortality among men in the first and second National Health and Nutrition Examination Surveys (NHANES I and NHANES II) follow-up studies. Int J Obes Relat Metab Disord.

[B32] Heitmann BL, Erikson H, Ellsinger BM, Mikkelsen KL, Larsson B (2000). Mortality associated with body fat, fat-free mass and body mass index among 60-year-old swedish men-a 22-year follow-up. The study of men born in 1913. Int J Obes Relat Metab Disord.

[B33] Oppert JM, Charles MA, Thibult N, Guy-Grand B, Eschwege E, Ducimetiere P (2002). Anthropometric estimates of muscle and fat mass in relation to cardiac and cancer mortality in men: the Paris Prospective Study. Am J Clin Nutr.

[B34] Wiebe CG, Gledhill N, Jamnik VK, Ferguson S (1999). Exercise cardiac function in young through elderly endurance trained women. Med Sci Sports Exerc.

[B35] Astrand I, Astrand PO, Hallback I, Kilbom A (1973). Reduction in maximal oxygen uptake with age. J Appl Physiol.

[B36] Pollock ML, Foster C, Knapp D, Rod JL, Schmidt DH (1987). Effect of age and training on aerobic capacity and body composition of master athletes. J Appl Physiol.

[B37] Marti B, Howald H (1990). Long-term effects of physical training on aerobic capacity: controlled study of former elite athletes. J Appl Physiol.

[B38] Trappe SW, Costill DL, Vukovich MD, Jones J, Melham T (1996). Aging among elite distance runners: a 22-yr longitudinal study. J Appl Physiol.

[B39] Eskurza I, Donato AJ, Moreau KL, Seals DR, Tanaka H (2002). Changes in maximal aerobic capacity with age in endurance-trained women: 7-yr follow-up. J Appl Physiol.

